# The Effects of Live-Fire Drills on Visual and Auditory Cognitive Performance among Firefighters

**DOI:** 10.5334/aogh.2626

**Published:** 2020-11-17

**Authors:** Rasoul Hemmatjo, Mohammad Hajaghazadeh, Teimour Allahyari, Sajad Zare, Reza Kazemi

**Affiliations:** 1 Department of Occupational Health Engineering, School of Health, Urmia University of Medical Sciences, Urmia, IR; 2 Department of Occupational Health, School of Public Health, Kerman University of Medical Sciences, Kerman, IR; 3 Department of Ergonomics, School of Public Health, Shiraz University of Medical Sciences, IR

## Abstract

**Introduction:**

The nature of firefighters’ work is characterized by its unexpected emergencies, sleep deprivation, night shift schedules, and smoke exposure during firefighting.

**Methods:**

Eighteen firefighters were involved in simulated live-fire activities. Firefighters’ health status in terms of cardiovascular and mental conditions was checked by a physician and through reviewing their medical records. Firefighters’ cognitive functions were evaluated by visual and auditory continuous performance tests (VCPT and ACPT) and paced visual and auditory serial addition tests (PVSAT and PASAT).

**Results:**

VCPT and ACPT scores were lower after the activity relative to baseline. The results revealed that ACPT appears to be more difficult than VCPT. Also, PVSAT and PASAT scores decreased significantly after the experiment. PASAT scores following firefighting tasks experienced a more significant decline compared to those of the PVSAT.

**Conclusions:**

These findings suggest that firefighters have visual and auditory cognitive function problems following firefighting activities. In conclusion, auditory cognitive function was more influenced than the visual ability as a result of the experiment.

## 1. Introduction

Firefighters might be under notable stress in their work due to the great workload and psychological stressors during fire and rescue operations. Unlike many other professions, firefighters are engaged in an occupation in which they are exposed to several stressors (e.g., night shift schedules, sleep deprivation, sudden alarm calls, strenuous physical work, exposure to smoke and other harmful substances during fire suppression, heartbreaking and tragic incidents, and victim search and rescue operations) for an unforeseeable amount of time [1,2,3,4,5]. For the same reason, firefighting is an occupation that imposes high physiological and psychological stress on firefighters.

Nonetheless, information on the impact of live fire activities on firefighters’ cognitive function are very limited and only a few studies have concentrated on cognitive function changes in simulated typical firefighting activities. Previous projects have shown that simulated typical firefighting activities have an effect on cognitive function [6,7,8]. They have also revealed that simulated firefighting tasks enhance the impairment and increase of errors made during the cognitive function test [9]. Hence, in order to perform their tasks perfectly and prevent damages caused by fire suppression, firefighters must have certain physical and physiological abilities. On the other hand, firefighters must have specific cognitive abilities to communicate with command and other firefighters during firefighting operations and to remember different parts of the fire in the building full of smoke and heat in order to escape in an emergency. They should also be cognitively able to process data very fast, especially in search and rescue missions as well as stressful and daunting situations.

In previous research, visual tests have been employed to measure cognitive functions (e.g., sustain attention, working memory, and information processing) after firefighting activities [1,9,10]. The firefighting profession has a number of demanding responsibilities. For example, during firefighting and rescue operations, firefighters must constantly communicate with commanders and other fire service workers in emergency conditions in order to report the situation or to get help. In such stressful conditions, they should also search for victims and try to rescue those who are detained in different parts of the fire scene. To do so, they need to have the ability to clearly hear victims’ voices in the smoky and dark environments and to communicate with them in order to protect and save their lives and properties without delay and error. Fulfilling all these demanding responsibilities requires a firefighter to have good auditory cognitive function capacity.

Physical activity is an unavoidable part of every profession, and for firefighters it is influenced by weather conditions. Work participation in the heat for long periods can lead to heat injury, such as heat stroke, which is associated with impaired physical and mental performance, raising the possibility of work-related accidents [11,12]. However, previous research has neglected the impact of live-fire activities on firefighters’ auditory cognitive function. In this study we investigate the effect of live-fire operations on visual and auditory cognitive functions. It was supposed that different types of firefighting tasks during live-fire exposure would have an effect on cognitive functions, including attention and information processing performance, while wearing firefighting protective clothing.

## 2. Method

### 2.1. Participants

The participants were 18 healthy and professional firefighters. Before conducting the study, participants were familiarized with the experimental goals and procedures and given written informed agreement to participate. Firefighters’ health status in terms of cardiovascular and mental conditions were checked by a physician and by reviewing their medical records. Firefighters were not engaged in the study if they had any cardiovascular and mental disease or vision and hearing problems. Based on the results of this checkup, fit and healthy firefighters were recruited for participation in this investigation. The mean ± SD physical characteristics of the participants were as follows: age 31.46 ± 5.17 years, height 1.79 ± 0.06 m, weight 79.77 ± 12.72 kg, body mass index 24.56 ± 2.55 kg/m^2^, and body surface area 1.99 ± 0.18 m^2^.

### 2.2. Simulated firefighting tasks

**Figure 1:**
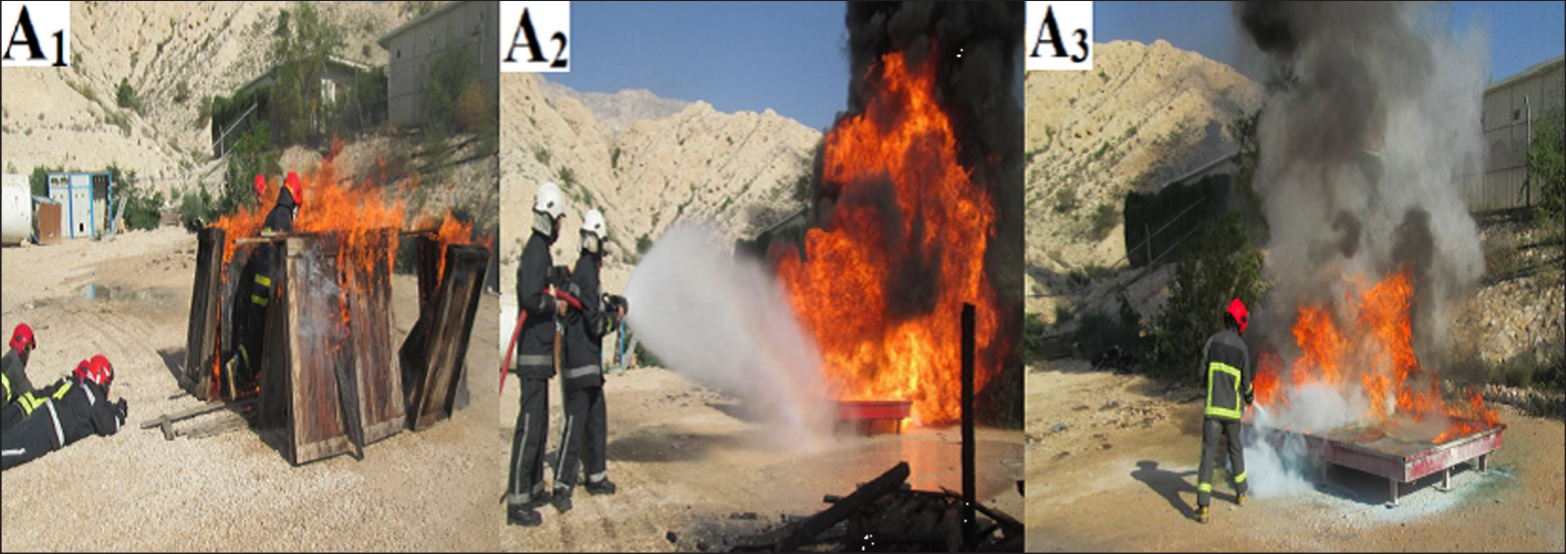
**A1)** Passing through the live-fire, **A2)** Extinguishing fire using water, **A3)** Shutting off fire with fire extinguisher.

Firefighting activities require high levels of aerobic fitness, anaerobic capacity, and muscular strength and endurance; however, previous findings show that many firefighters do not have high aerobic or anaerobic capacity [13,14]. Commonly, fire and rescue operations are composed of aerobic and anaerobic metabolism, along with remarkable muscular strength and endurance demands [15,16]. Based on former research, firefighters perform multiple aerobic and anaerobic tasks at high intensities during fire and rescue operations. Therefore, the current study simulated various types of live-fire drills duties, such as passing through fire, extinguishing fire, and rescue operations, which require high levels of aerobic fitness, anaerobic capacity, and muscular strength and endurance. These various types of firefighting activities were selected based on the recommendations provided by the firefighter’s commander and previous studies [3,17,18]. All firefighters participated in various types of simulated firefighting tasks; firefighters carried out live-fire suppression in a wide and open place. The suite of live-fire tasks included 1) passing through live fire, 2) extinguishing fire using water, 3) shutting off fire with fire extinguisher (Figure 1).

### 2.3. Cognitive function tests

#### 2.3.1. Visual and auditory continuous performance tests (VCPT and ACPT)

Visual and auditory continuous performance tests aim at measuring the capacity of sustained attention [19,20,21]. The CPT was initially introduced by Rosvold et al. and was expanded later when investigators started using a wide diversity of presentation ways, including auditory and visual presentations [22]. In this study, we used both visual continuous performance test (VCPT) and auditory continuous performance test (ACPT). The visual continuous performance test (VCPT) assesses both the visual reaction time (VRT) and the visual correct response (VCR). The auditory continuous performance test (ACPT) measures both the auditory reaction time (ART) and the auditory correct response (ACR).

In line with previous studies, in order to conduct the cognitive function test, participants were seated at a 15–24 inch distance from the computer monitor. The center of the monitor was 1–2 inches below eye level. A two-button ergonomic mouse placed in front of the computer screen was used to record participants’ responses; that is, they should press the left button of the mouse. The stimuli included ten pictures, with each of them being shown on a computer screen for 200 milliseconds. The visual stimuli (i.e., a star shape) were yellow in color, as high as 1.5 inches, and displayed with a frequency of 20%. In the VCPT test, firefighters were asked to immediately press a button if they saw the star shape on the screen. In the ACPT, auditory stimuli (i.e., ‘a star’) were presented with standard headphones. The computer screen was blank during auditory presentation. In the ACPT test, participants were instructed to immediately press a button if they heard the star name from the headphone. This test lasted for about 4 minutes, during which 150 stimuli were presented to the participants.

#### 2.3.2. Paced Visual and Auditory Serial Addition Tests (PVSAT and PASAT)

The paced auditory serial addition test (PASAT) is often used to evaluate information processing and working memory [23,24]. A visual version of the cognitive test (paced visual serial addition test or PVSAT) has also been introduced to examine information processing and working memory [25,26]. In this study, both Paced Visual Serial Addition Tests (PVSAT) and Paced Auditory Serial Addition Tests (PASAT) modalities were used.

Following the lead of previous research, in order to conduct the PVSAT and PASAT tests, firefighters should be seated in front of the computer monitor and wear headphones. In PASAT, participants are invited to listen to 61 single digit numbers (varying from 1 to 9), with an interval of 3 seconds between every two numbers. They should then calculate the sum of the last two numbers they have heard from the headphone. For instance, if 3 and 7 are presented consecutively, the correct response is 10. The participants should calculate the sum of the two digits before the following one is presented to them; otherwise, it will not be regarded as a correct response. The maximum score that participants can obtain in this test (based on the number of correct responses) is 60. The rectangle on the computer screen is blank during auditory presentation. PVSAT is very similar to PASAT, with the only difference that the 61 numbers from 1 to 9 are presented on the monitor. The participants should sum each number with the preceding digit and vocalize the sum. In both PVSAT and PASAT tests, firefighters are asked to say the summations loudly into a microphone to estimate the correct responses. This test lasted for about 3 minutes.

### 2.4. Experimental design

During simulated firefighting and life-saving operations, participants wore firefighting protective clothing (FPC), protective gloves, and a protective helmet and boots. The total weight of the personal protective equipment (PPE) was 15 to 19 kg. To examine the effects of simulated firefighting and rescue operations on sustained attention, information processing, and working memory, all firefighters performed visual and auditory cognitive function tests before and after conducting firefighting activities.

After the whole familiarization experiment, all firefighters performed the defined tasks: (1) to examine the effect of live-fire drills on cognitive function (sustained attention) by use of the Visual Cognitive Performance Test (VCPT), (2) to examine the effect of live-fire drills on cognitive function (sustained attention) by use of the Auditory Cognitive Performance Test (ACPT), (3) to examine the effect of live-fire drills on cognitive function (information processing and working memory) by the use of the Paced Visual Serial Addition Test (PVSAT), (4) to examine the effect of live-fire drills on cognitive function (information processing and working memory) by the use of the Paced Auditory Serial Addition Test (PASAT).

In line with the protocol, all firefighters initially accomplished the visual and auditory cognitive function tests in the control room (12 m^2^ indoor space, WBGT 22°C and 50% relative humidity) before live-fire drills; each firefighter performed defined live-fire drills for about 30 minutes. They were asked to perform the drill intently and without competing. After that, they again accomplished visual and auditory cognitive function tests in the control room. It took about 45–50 minutes for each firefighter to finish all defined live-fire drills and the cognitive function test.

### 2.5. Statistical analysis

The collected data were fed into Statistical Package for the Social Sciences (SPSS) 21 (SPSS Inc., Chicago, IL, USA). First, Kolmogorov-Smirnov (KS) test was run to evaluate the normal distribution of the data. After that, a paired samples t-test was conducted to compare cognitive function responses for both visual and auditory tests before and after performing the firefighting tasks (P < 0.05).

## 3. Results

### 3.1. Visual and auditory continuous performance tests (VCPT and ACPT)

**Figure 2:**
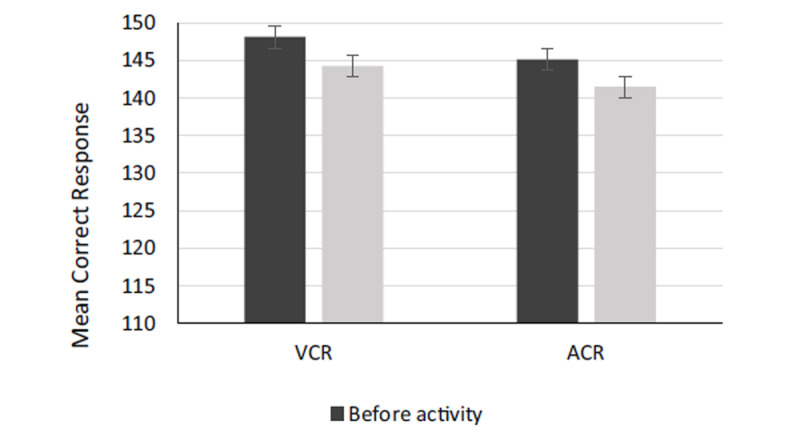
Mean VCR and ACR scores before and after the experiment. VCR and ACR stand for visual correct response (VCR) and auditory correct response (ACR) in the continuous performance test.

The results of the VRT and ART before and after the live-fire activities are displayed in Figure 2. The mean scores of VRT and ART were (408.00 ± 5.70_msec), (390.89 ± 6.14_msec), (396.00 ± 5.30_msec), and (379.22 ± 5.16_msec) for before-VRT, after-VRT, before-ART, and after-ART, respectively. For both visual VRT and auditory ART, there was a significant decrease following live-fire tasks relative to the baseline.

**Figure 3:**
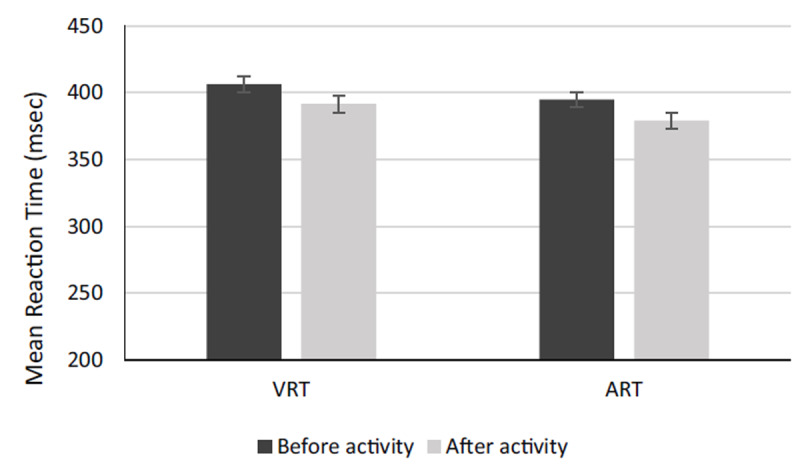
Means of VRT and ART scores before and after the experiment. VRT and ART stand for visual reaction time (VRT) and auditory reaction time (ART) in the continuous performance test.

Figure 3 depicts the impact of simulated live-fire tasks on visual and auditory response accuracy or correct response. Mean scores of VCR and ACR were (148.06 ± 1.21_CR), (144.06 ± 1.43_CR), (145.11 ± 1.27_CR), and (141.44 ± 1.91_CR) for before-VCR, after-VCR, before-ACR, and after-ACR, respectively. For all firefighters, VCR and ACR significantly decreased relative to the baseline during simulated live-fire activities. As observed in Figure 3, it can be said that the VCR and ACR were significantly lower after the experiment compared with before activity.

Paired comparisons of VRT and ART before and after the firefighting work are presented in Table 1. The results of paired samples t-tests showed a significant difference in VRT and ART between before and after activity phases (P < 0.05). The results also demonstrated that there is a significant mean difference between VRT and ART after performing live-fire tasks (P < 0.05). More precisely, ART (11.56 point) was faster than VRT after performing live-fire activities (see Table 1). The results of paired samples t-tests further revealed a significant difference in the VCR and ACR between before and after activity phases (P < 0.05) (Table 1). Moreover, based on Table 1 there is a significant difference between VCR and ACR after performing live-fire tasks (P < 0.05). In other words, ACR experienced a significantly bigger decline (2.61 point) after performing live-fire tasks compared with VCR (P < 0.05). According to the results, auditory cognitive function was more influenced than the visual cognitive function.

**Table 1:** Paired comparison of visual continuous performance test (VCPT) and auditory continuous performance test (ACPT) before and after the experiment.

Cognitive function test	Mean Difference	SE	*p* value

(before -VRT vs. after -VRT)	17.11	2.31	<0.05
(before -ART vs. after -ART)	16.77	2.04	<0.05
(after -VRT vs. after -ART)	11.66	0.90	<0.05
(before -VCR vs. after -VCR)	4.00	0.35	<0.05
(before -ACR vs. after -ACR)	3.66	0.45	<0.05
(after -VCR vs. after -ACR)	2.61	0.25	<0.05

*Note*: SE = standard error; VCR = visual correct response; VRT = visual reaction time; ACR = auditory correct response; ART = auditory reaction rime.

### 3.2. Paced visual and auditory serial addition tests (PVSAT and PASAT)

**Figure 4:**
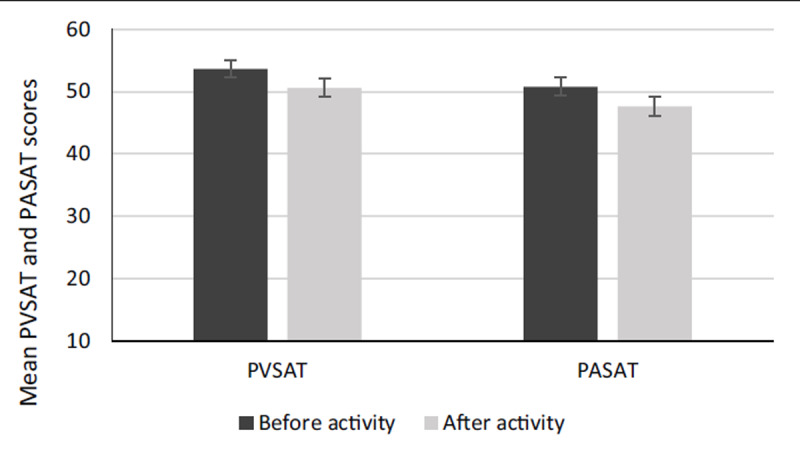
Means of PVSAT and PASAT scores before and after the experiment.

The results of the PVSAT and PASAT tests before and after activity can be seen in Figure 4. Mean PVSAT and PASAT scores (correct response) were (53.17 ± 1.24), (50.11 ± 1.18), (50.56 ± 1.38), and (47.33 ± 1.13) for before-PVSAT, after-PVSAT, before-PASAT, and after-PASAT, respectively. As shown in Figure 4, the firefighters scored significantly lower following live-fire operations than the before activity on the PVSAT and PASAT tests.

Table 2 describes the paired comparison of PVSAT and PASAT before and after the firefighting work. Paired samples t-test revealed a significant difference between the before and after activity in both PVSAT and PASAT (P < 0.05). The aim was to investigate whether there was any measurable difference between the visual (PVSAT) and auditory (PASAT) tests following live-fire activities (P < 0.05). According to Table 2, there is a significant mean difference between visual (PVSAT) and auditory (PASAT) after performing firefighting tasks (P < 0.05). In other words, PASAT scores following live-fire tasks experienced a more significant decline (2.77 point) compared to those of the PVSAT.

**Table 2:** Paired comparison of paced visual serial addition test (PVSAT) and paced auditory serial addition test (PASAT) before and after the experiment.

Cognitive function test	Mean Difference	SE	*P* value

(before -PVSAT vs. after -PVSAT)	3.05	0.23	<0.05
(before -PASAT vs. after -PASAT)	3.22	0.26	<0.05
(after -PVSAT vs. after -PASAT)	2.77	0.20	<0.05

*Note*: SE = standard error.

## 4. Discussion

As one of the first attempts, we investigated firefighters’ cognitive functions with visual and auditory cognitive function tests before and after the simulated live-fire. According to former studies, the specific characteristics of firefighting protective clothing (i.e., being heavy, thick, and massive) limit the degree of water evaporation and raise metabolism [27,28]. This causes disturbances in the regulation of body temperature, a phenomenon that leads to heat storage and can also cause impairments in the firefighters’ cognitive function [29]. Outside the firefighting operations, Neave et al. investigated the effects of protective helmets on cognitive function and demonstrated that wearing a protective helmet led to some cognitive function impairments in sustained attention and reaction times [30]. During firefighting operations, the stress level goes up, making it necessary for the body to deal with it. This may cause a reduction in the capacity of information processing and decision-making [27]. This can be explained in light of the stress hormones that are secreted as a result of various stressors; that is, stressful situations lead to the production of steroids, which can easily overcome the blood–brain barrier and reach the brain. They can then influence learning and memory by binding to receptors located in different areas of the brain that are responsible for learning and memory [31].

In the present study, all firefighters showed significantly shorter VRT and ART following firefighting activity when compared with the before-activity performance. These firefighters also had a significantly lower VCR and ACR after experiment compared with the before experiment. ACPT appears more difficult than VCPT, with the mean ART and ACR scores being (11.56) and (2.61) points lower than those of the VRT and VCR, respectively. McMorris et al. concluded that when plasma norepinephrine concentrations rise, the number of errors committed on a flanker task during heavy exercise goes up as well; nonetheless, the greater the increases in epinephrine and adrenocorticotropic hormone (ACTH) concentrations, the better the reaction time. Given that norepinephrine, epinephrine, and ACTH show increased arousal, it may be hypothesized that the speed and accuracy components of the cognitive function task were affected in different ways [32].

The results of this study indicate the potential attention impairment in both visual and auditory CPT. Because the CPT test is used to evaluate sustained attention, it can be argued that decreases in CPT scores following firefighting activity demonstrate impairment in sustained attention. According to the results, it can also be concluded that auditory attention has a more profound impact following the experiment. Because of the existing lacuna in the literature, little is known about alterations in visual and auditory sustained attention following firefighting activities. Previous research on areas other than fire and rescue service reported that in a group of normal controls, higher correct responses were found in the visual CPT when compared to an auditory CPT. The study also listed a difference between auditory ACPT and visual VCPT scores [33].

The results of the current study support previous findings, which have shown that visual reaction time decreases after firefighting activities. Prior research has revealed that firefighters achieve faster visual reaction time as a result of simulated firefighting activities [1]. Zhang et al. found faster visual reaction time following treadmill exercise in the environmental chamber [8]. There is evidence that performing multiple activities may decrease the number of correct responses in a continuous performance test [34].

In this research, we also evaluated information processing and working memory before and after the experiment among firefighters by administering PVSAT and PASAT. It has been discovered that the PASAT test is difficult for healthy subjects [35,36]. As expected, the results of the present study showed decrements in both PVSAT and PASAT scores after performing firefighting activities as compared with before activity. The present study demonstrated a greater rate of reduction in the auditory PASAT versus visual PVSAT modality among the firefighters following experiment. There was a mean difference of 2.81 in total scores (number of correct responses) between the visual PVSAT and auditory PASAT. These findings demonstrated that firefighters have information processing and working memory problems following firefighting and rescue operations. These problems influence visual and auditory states and may cause dangers in work performance. The results of PVSAT and PASAT are in line with previous research findings demonstrating impairment on this cognitive function test. For example, after evaluating information processing speed in 74 college students, Fos et al. reported that the participants’ PASAT scores were lower than their PVAST scores [37]. According to Razjouyan et al., subjects have better performance in PVSAT than PASAT and the number of correct responses in PVSAT (54.40 ± 7.03) is higher than that in PASAT (48.90 ± 6.42) [38]. Another study examined 10 healthy volunteers’ cognitive function changes after 50 minutes of treadmill exercise while wearing thermal protective clothing in a heated room (33–35°C), and they reported declines in working memory and reaction time following 60 and 120 minutes after the exercise [39]. Finally, the literature review shows there is little information about multiple firefighting activity impact on visual and auditory cognitive function. The present study tried to disclose the effects of live-fire drills on visual and auditory cognitive function in the simulated firefighting conditions.

## 5. Conclusion

These findings demonstrate that physical activity during fire and rescue operations have a significant impact on a firefighter’s attention, information processing, and working memory, which are extended to visual and auditory states. According to the findings, it can be concluded that auditory attention, information processing, and working memory were more deteriorated than visual ability as a result of the experiment. These cognitive function problems may cause difficulties in work performance. Based on the results of the current study, it is recommended that policy makers allocate time for useful methods (e.g., body cooling, breaking times, and replacement of exhausted personnel with fresh ones) to improve firefighters’ visual and auditory cognitive functions during firefighting and rescue operations.
